# An anthrax toxin variant with an improved activity in tumor targeting

**DOI:** 10.1038/srep16267

**Published:** 2015-11-20

**Authors:** Alexander N. Wein, Diane E. Peters, Zaheer Valivullah, Benjamin J. Hoover, Aparna Tatineni, Qian Ma, Rasem Fattah, Thomas H. Bugge, Stephen H. Leppla, Shihui Liu

**Affiliations:** 1 Microbial Pathogenesis Section, Laboratory of Parasitic Diseases, National Institute of Allergy and Infectious Diseases, National Institutes of Health, Bethesda, Maryland, USA; 2 Proteases and Tissue Remodeling Section, Oral and Pharyngeal Cancer Branch, National Institute of Dental and Craniofacial Research, National Institutes of Health, Bethesda, Maryland, USA; 3 Program of Pharmacology and Experimental Therapeutics, Tufts University School of Medicine, Boston, MA, USA

## Abstract

Anthrax lethal toxin (LT) is an A-B type toxin secreted by *Bacillus anthracis*, consisting of the cellular binding moiety, protective antigen (PA), and the catalytic moiety, lethal factor (LF). To target cells, PA binds to cell-surface receptors and is then proteolytically processed forming a LF-binding competent PA oligomer where each LF binding site is comprised of three subsites on two adjacent PA monomers. We previously generated PA-U2-R200A, a urokinase-activated PA variant with LF-binding subsite II residue Arg200 mutated to Ala, and PA-L1-I210A, a matrix metalloproteinase-activated PA variant with subsite III residue Ile210 mutated to Ala. PA-U2-R200A and PA-L1-I210A displayed reduced cytotoxicity when used singly. However, when combined, they formed LF-binding competent heterogeneous oligomers by intermolecular complementation, and achieved high specificity in tumor targeting. Nevertheless, each of these proteins, in particular PA-L1-I210A, retained residual LF-binding ability. In this work, we screened a library containing all possible amino acid substitutions for LF-binding site to find variants with activity strictly dependent upon intermolecular complementation. PA-I207R was identified as an excellent replacement for the original clockwise-side variant, PA-I210A. Consequently, the new combination of PA-L1-I207R and PA-U2-R200A showed potent anti-tumor activity and low toxicity, exceeding the performance of the original combination, and warranting further investigation.

Anthrax lethal toxin (LT), secreted by *Bacillus anthracis*, consists of two polypeptides: the cellular binding moiety protective antigen (PA, 83 kDa) and the enzymatic moiety lethal factor (LF, 90 kDa)^1^. To target mammalian cells, PA binds to tumor endothelial marker 8 (TEM8) or capillary morphogenesis protein 2 (CMG2)^2^,^3^ (also known as anthrax toxin receptors 1 and 2) on the cell membrane and is cleaved after the sequence RKKR by the protease furin on the cell surface^4^. This releases the N-terminal 20-kDa fragment (PA_20_), which allows for oligomerization of the activated, membrane-bound 63-kDa fragment PA_63_ into a heptamer or octamer. The PA oligomers have binding sites for LF that span adjacent monomers^5^,^6^. Three or four molecules of LF bind to the PA oligomers and the complexes are then endocytosed, after which a pH change within the endosomes causes the PA oligomer to form a pore in the endosomal membrane, allowing LF to translocate into the cytosol. LF is a zinc metalloprotease that enzymatically inactivates mitogen-activated protein kinase kinases, shutting down the ERK, p38, and JNK pathways^7^,^8^, leading to the death of experimental animals^9^,^10^,^11^.

Since proteolytic activation of PA on the cell-surface is absolutely required for the action of the toxin, modification of the cleavage site amino acid sequence can create tumor-selective PA variants that are only activated in the presence of tumor-associated proteases. Two such PA variants are PA-L1, which is activated by matrix metalloproteinases (MMPs)^12^,^13^, and PA-U2^14^,^15^,^16^, which is activated by urokinase plasminogen activator (uPA). Because malignant tissues overexpress MMPs and uPA^17^,^18^,^19^, these mutant toxins are preferentially activated in tumors and demonstrate significant antineoplastic potential. Based on the fact that each LF-binding site is formed by adjacent PA_63_ monomers, we generated intermolecular complementing PA variants that require the presence of both MMPs and uPA for activation^16^. Three subsites have been identified on the surface of the PA oligomer that are critical for LF binding (Fig. 1A)^5^,^6^,^20^. Subsites I and III are on the clockwise-side monomer; subsite I consists of residue R178 and subsite III includes residues I207, I210, and K214. Subsite II is on the counterclockwise-side monomer and comprises residue R200. Mutations at these sites disrupt LF binding and result in diminished toxicity. Specifically, we found that alanine mutations at R200 in PA-U2 (resulting in PA-U2-R200A) and I210 in PA-L1 (resulting in PA-L1-I210A) disrupted the toxicity of the PA oligomer^16^. However, the heterogeneous oligomer formed by co-administration of PA-U2-R200A and PA-L1-I210A restored LF binding capacity by intermolecular complementation, forming a toxin system requiring simultaneous presence of both tumor-associated proteases for activation^16^,^21^. However, PA-U2-R200A, and in particular PA-L1-I210A, each individually retained some residual LF-binding ability^16^. In this work, we screened a PA variant library containing all possible substitutions at residues R200, R178, I207, I210, and K214 to find PA variants with activities that were strictly dependent on intermolecular complementation and on the presence of the two distinct tumor-associated proteases to achieve highly specific tumor targeting.

## Results

### Mutagenesis and expression of PA mutants

**Figure 1:**
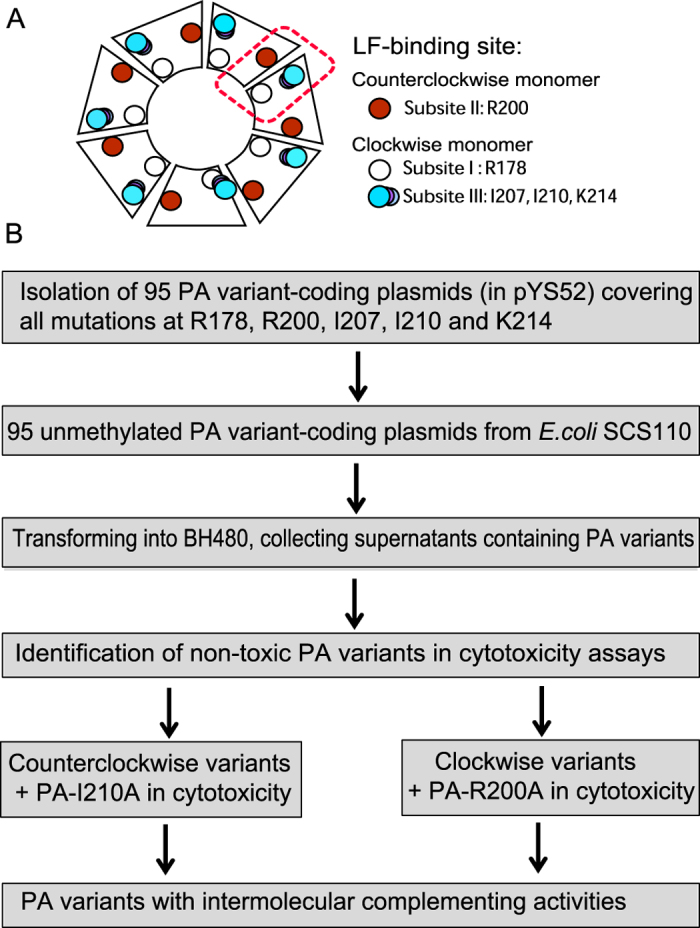
Screening for improved intermolecular complementing PA variants. (**A**) Schematic representation of PA oligomers, with each LF-binding site consisting of 3 subsites located on adjacent PA monomers. While a heptamer is shown, the same interactions occur within octamers. (**B**) Screening workflow to select improved intermolecular complementing PA variants. Unmethylated expression plasmids were isolated from dam^−^ and dcm^−^ *E. coli* SCS110, because only unmethylated plasmids are able to transform *B. anthracis.* BH480 transformants were grown and supernates assayed for protein expression and activity.

To select PA variants with activity strictly dependent on intermolecular complementation for LF-binding, we constructed a PA variant library with NNS (N = any nucleotide, S = C or G) codons at LF-binding subsite residue R200 on the counterclockwise-side monomer, and the residues R178, I207, I210, and K214 on the clockwise-side monomer (Fig. 1) (also refer to Fig. 3A in^6^). While DNA coding sequences for all the possible PA variants were isolated, some proteins were not expressed, or were expressed only at very low levels, from the corresponding transformed avirulent *B. anthracis* BH480 strains. Out of a theoretical library of 95 mutants, 79 were successfully expressed and supernatants containing the secreted PA variants were prepared for initial testing. PA proteins were usually expressed at high levels by the correspondingly transformed BH480 strains, often reaching levels above 50% of the total protein in the culture supernatant. Of the sixteen proteins that were not expressed, nine were mutants of K214 (C, D, E, F, G, P, S, W, Y), four were mutants at I207 (D, K, P, S), two were mutants of I210 (G, N), and one was an R178 mutant (P). K214 lies at the end of a α-helix and the side chain makes no close contact with any other residues on PA. It is not clear why so many mutants at this position could not be expressed.

### PA-I207R is an improved clockwise-side monomer variant

The sterilized supernatants containing PA proteins were screened for activity relative to the original versions of the intermolecular complementing, PA-R200A or PA-I210A, as appropriate using cytotoxicity assays. In the screen of counterclockwise-side monomer mutants, ten mutant PA-R200X proteins (C, D, E, G, I, M, P, S, V, and W) were initially identified as being less intrinsically toxic than PA-R200A. However, later detailed characterization found that these PA variants also showed reduced intermolecular complementation with PA-I210, resulting in less LF-induced cytotoxicity. As an example, although PA-R200C was slightly less toxic than PA-R200A, it was also slightly less effective in complementing with PA-I210A to promote killing of RAW264.7 cells (data not shown). Therefore, none of the counterclockwise-side monomer PA variants were found to be significantly superior to the original PA-R200A.

**Figure 2:**
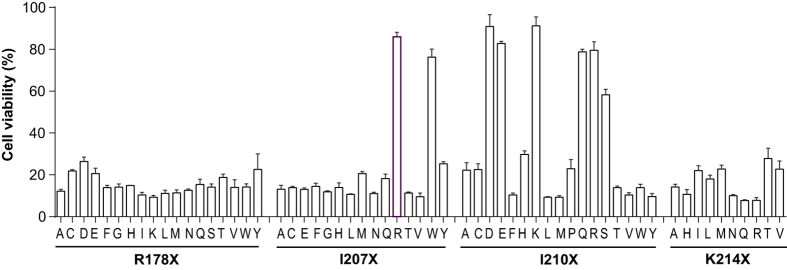
Cytotoxicities of the clockwise-side monomer PA variants. Supernatants containing PA variant proteins secreted from the corresponding transformed BH480 strains were prepared. The concentrations of PA variants were estimated by densitometry of stained gels. RAW264.7 cells were treated with the respective PA variants at ≈500 ng/mL in the presence of 100 ng/mL LF for 20 h. Cell viability was measured by MTT assay for the final hour as described in Methods. PA variants with cytotoxicity significantly lower than PA-I210A were selected for further analyses.

**Figure 3:**
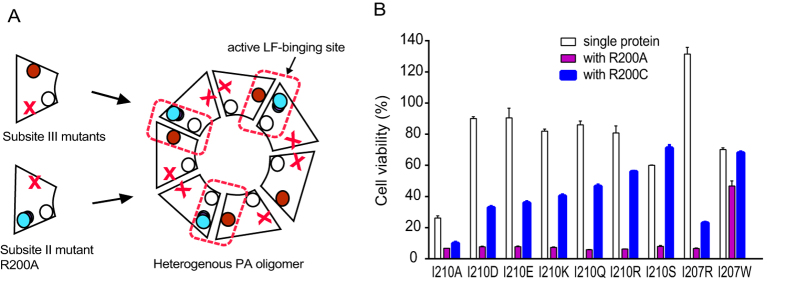
Intermolecular complementing activities of the PA variant candidates. (**A**) Schematic representation of intermolecular complementation of the PA variants. (**B**) RAW 264.7 macrophages were treated with PA variants at 500 ng/mL in the presence of 100 ng/mL LF or at 250 ng/mL plus 250 ng/mL PA-R200A or PA-R200C in the presence of 100 ng/mL LF for 6 h. Then cell viabilities were determined by MTT assay as described above.

In screens for the clockwise-side monomer variants PA-R178X, PA-I207X, PA-I210X, and PA-K214X, eight proteins with greatly reduced inherent cytotoxicity to RAW264.7 macrophages were identified: I207R, I207W, I210D, I210E, I210K, I210Q, I210R, and I210S (Fig. 2). These were tested in combination with R200A and R200C for their ability in intermolecular complementation (Fig. 3). All these clockwise-side mutants could be complemented by PA-R200A and to a lesser extent by PA-R200C. Among these PA variants, PA-I207R behaved the best in complementing with both PA-R200A and PA-R200C to achieve killing of RAW264.7 cells. Therefore, PA-I207R was identified as an improved clockwise-side monomer variant for the intermolecular complementation PA system, displaying very low cytotoxicity when used singly.

**Figure 4:**
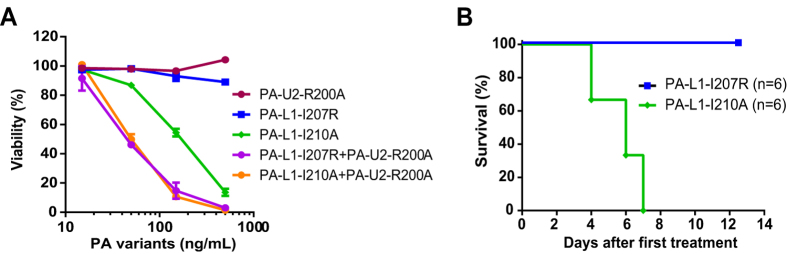
PA-L1-I207R is an improved clockwise-side PA variant having little to no toxicity. (**A**) Cytotoxicities of PA variants tested singly and in combination to B16-BL6 cells. Cells were incubated with the indicated PA variants in the presence of 30 ng/mL FP59 for 48 h. Cell viabilities were then determined by MTT assay as described above. (**B**) PA-L1-I207R shows improved safety to mice when used singly with FP59. C57BL/6J mice were injected intraperitoneally with 3 doses of 20 μg PA plus 10 μg FP59 at days 0, 1 and 2 and checked twice daily for two weeks for survival.

To further characterize PA-I207R, the MMP-activated variant, i.e., PA-L1-I207R was generated and the protein purified. The new combination of PA-L1-I207R and PA-U2-R200A was compared with the original combination of PA-L1-I210A and PA-U2-R200A for cytotoxicity towards mouse melanoma B16-BL6 cells, which express high levels of both MMPs and uPA. Intriguingly, PA-L1-I207R showed similar activity as PA-L1-I207A in complementing with PA-U2-R200A to kill B16-BL6 cells in the presence of FP59, a LF fusion effector protein that kills cell in a PA-dependent manner^22^ (Fig. 4A). Remarkably, the single component PA-L1-I207R showed no cytotoxicity whereas the original counterpart PA-L1-I210A displayed moderate cytotoxicity to B16-BL6 cells when used singly (IC_50_ = 200 ng/mL) in these assays (Fig. 4A). PA-U2-R200A also showed no cytotoxicity when used singly in these settings (Fig. 4A).

We further compared the toxicity of PA-L1-I207R and PA-L1-I210A when administered with FP59 to C57BL/6J mice. We found that PA-L1-I207R was much less toxic than PA-L1-I210A in that all the mice challenged with 3 doses of 20 μg PA-L1-I207R and 10 μg FP59 survived whereas all the mice challenged with three doses of 20 μg PA-L1-I210A and 10 μg FP59 succumbed to the challenges within a week (*P* = 0.0007, log-rank test (Mantel-Cox)) (Fig. 4B).

### The combination of PA-L1-I207R plus PA-U2-R200A shows high efficacy in antitumor activity

**Figure 5:**
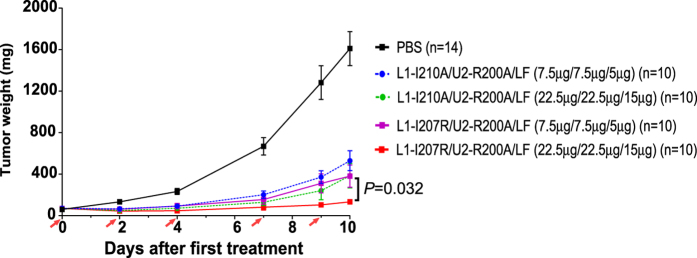
Potent anti-tumor activity and low toxicity of the PA-L1-I207R and PA-U2-R200A combination in the B16-BL6 syngeneic tumor model. B16-BL6 tumor-bearing mice in groups of 10 or 14 were treated intraperitoneally with PBS or with the original or new intercomplementing PA variant combinations at the doses shown. Fives doses were administered on the days shown by the red arrows. The new combination of PA-L1-I207R and PA-U2-R200A showed increased efficacy versus the original combination of PA-L1-I210A and PA-U2-R200A in inhibiting tumor growth. Data are shown with mean ± SEM. Student’s t test. Tumor weights were estimated using the formula: tumor weight (mg) = (length in mm × width in mm^2^) × 0.5.

To further evaluate the anti-tumor activity of the new PA variant, we performed a side-by-side comparison of the new combination, i.e., PA-L1-I207R plus PA-U2-R200A, and the original combination of PA-L1-I210A and PA-U2-R200A in treatment of B16-BL6 syngeneic tumors in C57BL/6J mice. The tumor-bearing mice were treated with either PBS, low doses of the new combination or the original combination of the PA variants plus LF (7.5 μg/7.5 μg/5 μg), or high doses of each combination plus LF (22.5 μg/22.5 μg/15 μg). All of the toxin-treated groups showed significant anti-tumor activities compared to the PBS control group (Fig. 5) (P < 0.0001 for all toxin-treated groups, Student’s t test). Interestingly, the new combination of PA-L1-I207R and PA-U2-R200A showed significantly higher anti-tumor activity than the original combination of PA-L1-I210A and PA-U2-R200A (Fig. 5) (P = 0.0326 for the two high dose groups at day 10, Student’s t test). A mortality of 20% was observed in the PBS group before termination of the experiment at day 10, with deaths apparently due to the high tumor burden. The groups receiving the low doses of both combinations had 10% mortality, whereas in the high dose groups the new combination appeared to the safer, with a10% mortality versus 30% mortality in the original combination group (although this difference was not statistically significant).

## Discussion

Anthrax toxin, one of the best characterized protein delivery systems, has been reengineered for tumor targeting^23^. The PA protein can be modified in multiple ways to achieve high specificity for tumors^21^,^24^,^25^,^26^,^27^. We have previously demonstrated that PA protease activation specificities can be redirected to tumor-associated proteases by replacing the amino acid sequence recognized by furin with sequences recognized by MMPs and uPA. The resulting PA variants, such as the MMP-activated PA-L1 and the uPA-activated PA-U2, can be used to selectively deliver effector proteins, LF and LF fusions, into tumor tissues, thereby producing strong cytotoxic action against a wide range of solid tumor types^13^,^14^,^16^,^21^,^24^,^25^. Interestingly, each LF-binding site on the PA oligomer bridges two adjacent PA monomers^5^,^6^,^20^. To exploit this feature in designing tumor-selective reagents, we previously generated a PA intermolecular complementation system consisting of PA-L1-I210A and PA-U2-R200A, in which toxin activation was dependent on the simultaneous presence of both MMPs and uPA^16^. These intermolecular complementing PA proteins showed high specificity for tumor targeting^13^,^21^. However, the PA-L1-I210A and PA-U2-R200A proteins still retained some innate LF-binding ability, leading to residual cytotoxicity toward tissues having a single activating protease, either MMPs or uPA, rather than both. To obtain improved intermolecular complementing PA variants, here we have screened a PA variant library covering all possible mutations at the counterclockwise-side PA monomer residue R200 and the clockwise-side monomer residues R178, I207, I210, and K214. Analysis of the crystal structure of PA_63_ heptamer with LF reveals that PA residue R200 forms a hydrogen bond with the backbone oxygen of LF at E139 as well as a salt bridge with the side chain of E139^20^. R178 forms a hydrogen bond with the backbone oxygen of H42 on LF. I207 has close interactions with LF residues V232 (3.4 Å), L188 (3.8 Å), and Y223 (4.2 Å). I210 is in close contact with LF residues Y236 (3.9 Å) and L188 (3.4 Å). K214 forms a salt bridge with LF residue D184.

Among all the possible substitutions of the clockwise-side monomer residues, we found that the PA-I207R variant completely lost LF-binding ability while fully retaining the ability to complement with the counterclockwise-side monomer PA-R200A, thus qualifying as an excellent replacement for the original PA-I210A variant. Surprisingly, although all of the nineteen PA variants at the only clockwise-side monomer residue R200 could be produced and compared, none of them were found to be better than the original PA-R200A. Although some of the variants at this position showed reduced toxicity when used singly, they also lost their complementing ability. These results suggested that the PA residue R200 plays an important role in maintaining the structural integrity of PA, so that only a limited number of substitutions could be tolerated at this position. Therefore, PA-L1-I207R was used to replace the original PA-L1-I210A to form an improved version of the intermolecular complementing toxin pair with PA-U2-R200A. Because the residual activity of the original pair (PA-L1-I210A and PA-U2-R200A) is mostly resultant from PA-L1-I210A^16^,^28^, this new combination represents a significant improvement over the original version.

The new pairing of PA-L1-I207R and PA-U2-R200A had a cytotoxic potency similar to that of the original combination toward B16-BL6 melanoma cells (Fig. 4A). Interestingly, the new version showed a higher anti-tumor activity in B16-BL6 tumors in mice. One possible explanation is that PA-L1–I207R may be structurally more stable than PA-L1-I210A so that higher amounts could reach tumor sites. We did observe that significantly lower yields of PA-L1–I210A were always obtained compared to that of PA-L1–I207R and wild type PA in our standard expression and purification procedure. This may result from a perturbation of the PA-L1-I210A structure due to the Ile^210^ to Ala mutation, making the protein more susceptible to protease degradation during purification. The new combination of the PA variants also seemed to have lower non-specific toxicity when used in the mouse tumor model. However, because of the low mortality rates (<30%) and the population sizes in this study (n = 10–14 for each group), this trend was not shown to be statistically significant.

In summary, the new combination of PA-L1-I207R and PA-U2-R200A showed potent anti-tumor activity and low toxicity, warranting further clinical development in conjunction with strategies to suppress an adaptive immune response as described recently^29^,^30^.

## Methods

### Mutagenic PCR

Mutations were introduced by overlap PCR with NNS codons into the plasmid pYS52 (a derivative of *B. anthracis* expression vector pYS5^31^), which contains a synthetic DNA sequence coding for domain II of PA with additional unique restriction sites. Phusion High-Fidelity DNA polymerase (New England Biologicals, Ipswich, MA) was used for mutagenic PCR reactions. Flanking primers used for all reactions were 5′-GTTAGACGATAAACCAGTCCT-3′ (PA-250) and 5′-CTACCAGATTTAAATCCTTTCCATTAAAAAT-3′ (Pa-SwaI-Rev). Specific primers were 5′-CAAGTGCTGGACCTACGGTTCCAGACNNSGACAATGATGGAATCCCTA-3′ (R178X mut), 5′-CTGGAACCGTAGGTCCAGCACTTG-3′ (R178X rev), 5′-CGAGGTAGAAGGATATACGGTTGATGTCAAAAATAAANNSACCTTTCTTTCACCATGG-3′ (R200X mut), 5′-TGACATCAACCGTATATCCTTCTACCTCG-3′ (R200X rev), 5′-CAAAAATAAAAGGACCTTTCTTTCAACATGGNNSTCTAATATTCATGAAAAGAAAGGG-3′ (I207X mut), 5′-CATGGTGAAAGAAAGGTCCTTTTATTTTTG-3′ (I207/210X rev), 5′-CAAAAATAAAAGGACCTTTCTTTCACCATGGATATCTAATNNSCATGAAAAGAAAGGG-3′ (I210X mut), 5′-CTTTCACCATGGATATCTAATATTCATGAAAAGNNSGGGTTAACCAAATATAAATCATC-3′ (K214X mut), and 5′-CTTTTCATGAATATTAGATATCCATGGTGAAAG-3′ (K214X rev). Mutagenic primers were combined with PA-SwaI-Rev and reverse primers with PA-250 for the first round of PCR. The products were gel purified and the complementary PCR fragments were extended to full length by 10 cycles of PCR before addition of PA-250 and PA-SwaI-rev and an additional 35 cycles. These mutagenic inserts were gel purified and the inserts, along with pYS52, were digested with PstI and HindIII. The digestion products were ligated overnight and transformed into chemically competent *E. coli* MC1061, which were plated on LB-agar plates containing 100 μg/mL ampicillin. Colony PCR using PA-250 and PA-SwaI-Rev primers was performed to identify positive clones. Colonies were screened using primer PA-250 to sequence the plasmid and identify clones having all possible substitutions. Selected clones were then grown overnight in LB broth containing 100 μg/mL ampicillin and the plasmids were extracted by mini-scale preparation.

### Expression of protein library

Mini-prepared plasmids from *E. coli* MC1061 were transformed into chemically competent *E. coli* SCS110, which is dam^−^ and dcm^−^. The purified, non-methylated plasmids from SCS110 were then transformed into an electrocompetent *B. anthracis* BH480 strain, which was plated on LB-agar containing 20 μg/mL kanamycin. BH480 is an avirulent large plasmids-cured, sporulation-defective *B. anthracis* strain with eight proteases deleted, serving as an efficient host for recombinant protein expression^32^. Single colonies were grown overnight in 5 mL FA medium containing 20 μg/mL kanamycin^32^. The supernatants containing the mutant PA proteins were sterilized by centrifugation and concentrated ~10 fold using Amicon Ultra-4 (30 K) Centrifugal Filter Devices (Millipore Corp., Billerica, MA). The supernatants were analyzed by native gel electrophoresis, strained with coommasie blue dye, and the protein concentrations were estimated by densitometry to compare the supernatant bands to a sample of purified PA. Some examples of these native gels were shown in Figure S1. This was performed twice on each protein.

### PA variant screen

RAW264.7 macrophages and murine melanoma B16-BL6 cells were grown in Dulbecco’s Modified Eagle Medium (Life Technologies, Grand Island, NY) supplemented with fetal bovine serum to 10% (Invitrogen) and gentamycin at 50 μg/mL (Invitrogen) at 37 °C in a tissue culture incubator with 5% CO_2_.

To test the PA variants for a loss of function, RAW 264.7 macrophages were plated in a 96-well plate at 10^5^ cells/well and grown overnight. The next day, the PA variants were added at a concentration of 500 ng/mL and LF at a concentration of 100 ng/mL. The cells were incubated for 20 h and MTT (3-(4,5-dimethyl-2-thiazolyl)-2,5-diphenyl-2H-tetrazolium bromide, Sigma, St. Louis, MO) was added at 500 μg/mL for the final hour. The medium was aspirated and the oxidized MTT was solubilized in 91% isopropanol containing 0.5% SDS and 0.038 M hydrochloric acid, then read at 570 nm using a SpectraMax 190 plate reader (Molecular Devices, Sunnyvale, CA). The absorbance was used to determine percent survival compared to an untreated control. PA variants that showed less toxicity than the original constructs PA-R200A and PA-I210A were subjected to a double-agent gain-of-function test. RAW 264.7 macrophages were seeded as above and respective PA variants (250 ng/mL) combined with PA-R200A or PA-I210A (250 ng/mL) and 100 ng/mL LF were added. The cells were incubated for 6 h and viability was measured as above.

### Creation and cytotoxicity of PA-L1-I207R

Site-directed mutagenesis was used to introduce mutation I207R into pYS5-L1ff, an overexpression plasmid for PA-L1, in which the furin cleavage sequence RKKR (residues 164-167) was replaced with a MMP substrate sequence GPLGMLSQ^12^). Primers used were 5′-AAAATAAAAGAACTTTTCTTTCACCATGGCGTTCTAATATTCATGAAAAGAAAGGATTA-3′ (I207R sense), 5′- TAATCCTTTCTTTTCATGAATATTAGAACGCCATGGTGAAAGAAAAGTTCTTTTATTTT-3′ (I207R antisense). Sense and anti-sense primers were used with the Quikchange Lightning Kit (Agilent, Santa Clara, CA) according to manufacturer’s recommendations and transformed into chemically competent *E. coli* XL-10 Gold. The plasmids were mini-prepared, sequenced, and transformed into *E. coli* SCS110 before transformation into BH480 for expression and purification. Cytotoxicities of PA-L1-I207R and PA-L1-I210A were tested singly and in combination with PA-U2-R200A (with the furin cleavage sequence replaced with an uPA substrate sequence PGSGRSA^15^) in the presence of 30 ng/mL FP59. B16-BL6 cells were incubated with the indicated toxins in 96-well plates for 48 h, and cell viabilities were determined by MTT assay as described above.

### Protein purification

PA-L1-I210A, PA-L1-I207R, PA-U2-R200A, LF and FP59 were expressed using pYS5-based expression plasmids from *B. anthracis* BH480 strain. The recombinant proteins secreted into culture supernatants were purified as described previously^32^,^33^. In brief, the expression plasmid transformed BH480 strains were grown in FA medium with 10 μg/ml kanamycin for 12 h at 37 °C. The proteins secreted into the culture supernatants were precipitated on Phenyl-Sepharose 6 Fast Flow resin (low substitution, GE Healthcare Life Sciences, Pittsburg, PA) (30 ml per liter supernatant) in the presence of 2 M ammonium sulfate in rotating bottles. The resin was collected on a porous plastic funnel and washed with wash buffer (1.5 M ammonium sulfate, 10 mM Tris-HCl (pH 8.0), 1 mM EDTA) and the proteins were eluted using elution buffer (0.3 M ammonium sulfate, 10 mM Tris-HCl (pH 8.0), 1 mM EDTA). The eluted proteins were precipitated by adding 2 M ammonium sulfate. The precipitate was collected by centrifugation, resuspended and dialyzed in 10 mM Tris-HCl (pH 8.0), 1 mM EDTA. The toxin proteins were further purified by chromatography on Q-Sepharose Fast Flow column (GE Healthcare Life Sciences) and eluted with a 0–0.5 M NaCl gradient in 20 mM Tris–HCl, 0.5 mM EDTA (pH 8.0). The toxin proteins were further purified by Sephacryl S-200 high resolution gel filtration (GE Healthcare Life Sciences) using 10 mM Tris-HCl (pH 8.0), 100 mM NaCl , and 0.5 mM EDTA to one prominent band at the expected molecular mass (please see Figure S2 as an example).

### *In vivo* toxicity of PA mutants

C57BL/6J male and female mice (10 to 12-week-old) were injected intraperitoneally with 20 μg PA-L1-I207R or PA-LI-I210A along with 10 μg FP59 at 0, 24, and 48 hours and checked twice daily for signs of malaise and mortality for two weeks following the first injection. Mice were euthanized at the end of experiment. All animal studies were carried out in accordance with protocols approved by the National Institute of Allergy and Infectious Diseases Animal Care and Use Committee.

### *In vivo* anti-tumor study

Twelve-week-old female C57BL/6J mice (Jackson Laboratory, Bar Harbor, Maine) were injected with 5 × 10^5^ B16-BL6 cells in the mid-scapular subcutis. B16-BL6 melanoma cells were a kind gift from Dr. Isaiah J. Fidler (MD Anderson Cancer Center, Houston, TX), and were authenticated by continual assessment of cellular morphology at both low and high magnifications. Eight days after injection, established tumors were measured with digital calipers (FV Fowler Company, Inc., Newton, MA). Tumor weights were estimated with the longest and shortest tumor dimensions in the formula: tumor weight (mg) = (length in mm × width in mm^2^) × 0.5^34^. Tumor-bearing mice were randomized into groups and injected intraperitoneally on study days 0 (eight days after tumor cell injection), 2, 4, 7, and 9 with a 200 μL PBS solution containing the PA variant proteins and LF, at doses shown in the figures. Mice were weighed and the tumors were measured before each injection. The study was ended on day 10 when tumors in the PBS alone control group reached 10% of the body weights, a condition requiring euthanasia according to the animal study protocol.

### Statistical analysis

Statistical analysis was done with unpaired student’s t test using Excel. Survival curves were analyzed using log-rank test (Mantel-Cox) using GraphPad Prism software.

## Additional Information

**How to cite this article**: Wein, A. N. *et al.* An anthrax toxin variant with an improved activity in tumor targeting. *Sci. Rep.* **5**, 16267; doi: 10.1038/srep16267 (2015).

## Supplementary Material

Supplementary Information
