# Supplementary material for: An anthrax toxin variant with an improved activity in tumor targeting
Source: Sci Rep. 2015 Nov 20;5:16267. doi: 10.1038/srep16267 (PMC4653645; doi:10.1038/srep16267)
Supplement: Supplementary Information [file srep16267-s1.pdf]

Supplementary Information

An anthrax toxin variant with an improved activity in tumor targeting

Alexander N. Wein[1]*, Diane E. Peters[2,3]*, Zaheer Valivullah[1]*, Benjamin J. Hoover[1], Aparna Tatineni[1], Qian Ma[1], Rasem Fattah[1], Thomas H. Bugge[2], Stephen H. Leppla[1], and Shihui Liu[1]

[1]Microbial Pathogenesis Section, Laboratory of Parasitic Diseases, National Institute of Allergy and Infectious Diseases, National Institutes of Health, Bethesda, Maryland, USA

[2]Proteases and Tissue Remodeling Section, Oral and Pharyngeal Cancer Branch, National Institute of Dental and Craniofacial Research, National Institutes of Health, Bethesda, Maryland, USA

[3]Program of Pharmacology and Experimental Therapeutics, Tufts University School of Medicine, Boston, MA, USA

*These authors contributed equally to this work

For correspondence: shliu@niaid.nih.gov, or sleppla@niaid.nih.gov

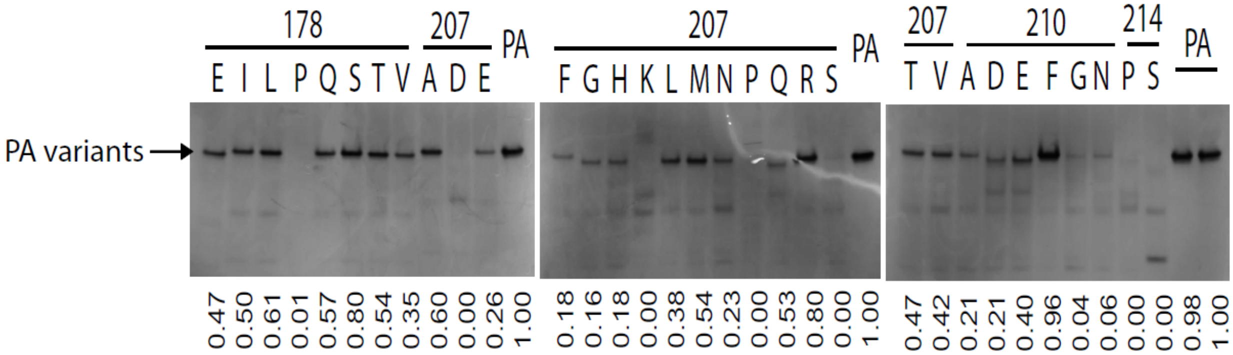

Figure S1. PA variant proteins concentrated from culture supernatants of the corresponding expressing BH480 strains.
The transformed BH480 strains were grown overnight in 5 mL FA medium containing 20 µg/mL kanamycin. The supernatants containing PA variant proteins were sterilized by centrifugation and concentrated 10-fold, and were analyzed by native gel electrophoresis. The protein concentrations were estimated by densitometry to compare the PA variant bands to a sample of purified PA with known concentration (the purified PA = 0.88 mg/mL). The relative concentrations of PA variant proteins to purified PA were given below of the images.

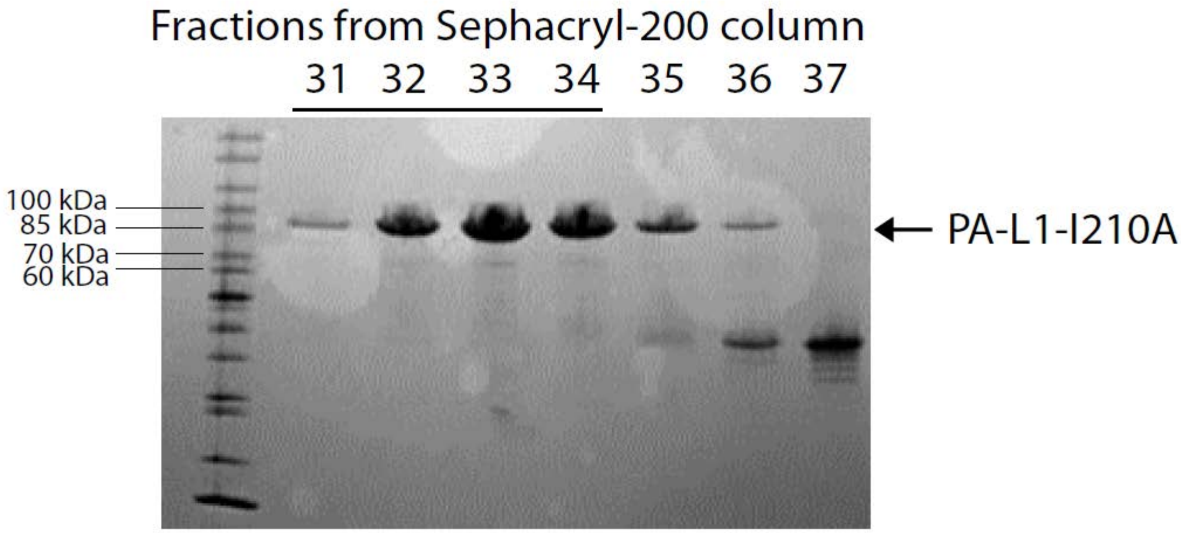

Figure S2. Fractions containing PA-L1-I210A eluted from the Sephacryl S-200 gel filtration column.

Partially purified PA-L1-I210A protein from chromatography on Q-Sepharose Fast Flow column (see Methods) was subjected to further purification by Sephacryl S-200 high resolution gel filtration. The fractions 31-34 with the major protein band of PA-L1-I210A shown on a SDS-PAGE gel were pooled, measured at A280, and stored at -80° as the final product.
